# Fimasartan-induced liver injury in a patient with no adverse reactions on other types of angiotensin II receptor blockers: A case report

**DOI:** 10.1097/MD.0000000000008905

**Published:** 2017-11-27

**Authors:** Dae Hwa Park, Gee Young Yun, Hyuk Soo Eun, Jong Seok Joo, Ju Seok Kim, Sun Hyung Kang, Hee Seok Moon, Eaum Seok Lee, Byung Seok Lee, Kyung Hee Kim, Seok Hyun Kim

**Affiliations:** a Department of Internal Medicine; b Department of Pathology, Chungnam National University Hospital, Chungnam National University School of Medicine, Daejeon, South Korea.

**Keywords:** angiotensin II receptor blockers, drug-induced liver injury, fimasartan

## Abstract

**Rationale:**

Angiotensin II receptor blockers (ARBs) are widely used for patients with hypertension, and fimasartan is a recently approved ARBs. Fimasartan can cause headache, dizziness, itching, and coughing. There have been several reports of hepatotoxicity in ARBs. However, there have not yet been published reports of the hepatotoxicity of fimasartan.

**Patient concerns:**

A 73-year-old man with hypertension experienced liver injury after fimasartan administration. He had a previous history of taking 3 types of ARBs each for more than 2 years before taking fimasartan, and there were no side effects on ARBs except for fimasartan.

**Diagnoses:**

Other factors that could cause liver injury were excluded in diagnostic tests, and fimasartan was suspected to be the causative agent.

**Intervention:**

Fimasartan was immediately discontinued and the patient was managed with supportive care via hepatotonics.

**Diagnoses:**

Other factors that could cause liver injury were excluded in diagnostic tests, and fimasartan was suspected to be the causative agent.

**Outcome:**

The liver injury due to fimasartan was confirmed by histology and accidental redosing.

**Lessons:**

We emphasize that liver function should be monitored during fimasartan administration because fimasartan may cause hepatotoxicity in patients who have no side effects with other types of ARBs. And fimasartan-induced liver injury may appear later than other ARBs.

## 1 Introduction

Angiotensin II receptor blockers (ARBs) are widely used drugs for patients with hypertension. The most recently approved ARB is fimasartan. In the clinical trial studies, fimasartan can cause side effects such as dizziness, headache, abdominal pain, nausea, palpitation, fatigue, diarrhea, and coughing. And there was limited published data of hepatotoxicity due to fimasartan.^[1]^ The first ARB used was losartan, and several reports have evaluated losartan-induced hepatotoxicity, although it is rare (<0.1%).^[2–4]^ Furthermore, there have been several other reports on irbesartan-, candesartan-, and valsartan-induced hepatotoxicity, which is far less common than losartan-induced hepatotoxicity.^[4–8]^ To our knowledge, there is no published report yet on liver injury caused by fimasartan. We report a case of hepatotoxicity caused by fimasartan. There are 3 peculiarities in this case. First, the patient had no side effects with other types of ARBs that had been taken before the drug was changed to fimasartan. Second, drug-induced hepatotoxicity occurred in this patient only 10 months after the first dose. Finally, after recovering from the first episode of hepatotoxicity that occurred, the patient was accidentally retaking fimasartan and the episode was recurred, so the causative agent was clearly identified.

## 2 Methods

Informed consent was obtained from the patient.

## 3 Case report

**Figure 1:**
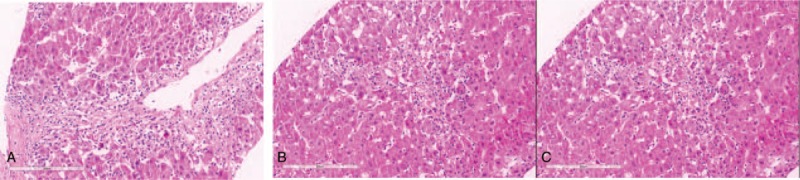
A and B, Liver biopsy showed hepatocellular necrosis in zones 2 and 3, with severe lobular inflammation. C, A liver biopsy showed spared periportal hepatocytes in zone 1 (hematoxylin and eosin: A: ×200, B: ×200, and C: ×200).

A 73-year-old South Korean man with hypertension was referred to our hospital from a local hospital due to elevated liver enzyme levels. Since July 14, 2002, he had been taking ramipril, which is an ACE inhibitor, as antihypertensive. As his physician changed, the medications were sequentially switched to candesartan 16 mg, ARB on June 24, 2010, irbesartan 300 mg on June 25, 2012, and telmisartan 80 mg on August 26, 2015. Then, the drug was switched to fimasartan 30 mg on September 16, 2015 and fimasartan 60 mg on October 7, 2015. He was referred from the local hospital and admitted to ours on August 4, 2016. He did not report any use of alcohol or illicit drugs. Blood work revealed acute liver dysfunction with an aspartate aminotransferase (AST) level of 233 U/L, alanine aminotransferase (ALT) level of 424 U/L, and international normalized ratio (INR) of 1.09. The total bilirubin, alkaline phosphatase (ALP), and gamma-glutamyl transpeptidase levels were 1.22 mg/dL, 112 U/L, and 118 U/L, respectively. The INR was in the normal range during the entire period that the patient took aspirin for nonvalvular atrial fibrillation. He had normal baseline laboratory results at the initiation of fimasartan administration. Hepatitis A, immunoglobulin M, and hepatitis B surface antigen were negative, and hepatitis C RNA levels were undetectable. Hepatitis E, immunoglobulin M, cytomegalovirus, immunoglobulin M, and Epstein–Barr virus viral-capsid antigen immunoglobulin M were also negative. However, the hepatitis B surface antibody was positive. Other etiologies, including autoimmune disease, common toxins, drugs, and iron- or copper-induced insults were considered. However, antimitochondrial, antismooth muscle, and antinuclear antibodies were all negative, and the serum copper, ceruloplasmin, and 24-hour urine copper levels were in the normal ranges. The modified Roussel Uclaf Causality Assessment Method (RUCAM) scale score was 8. These findings strongly suggested the presence of drug-induced liver injury. A percutaneous liver biopsy was performed, and hepatocellular necrosis was seen in zones 2 and 3, with spared periportal hepatocytes in zone 1 (Fig. 1), suggesting toxic hepatitis.

**Figure 2:**
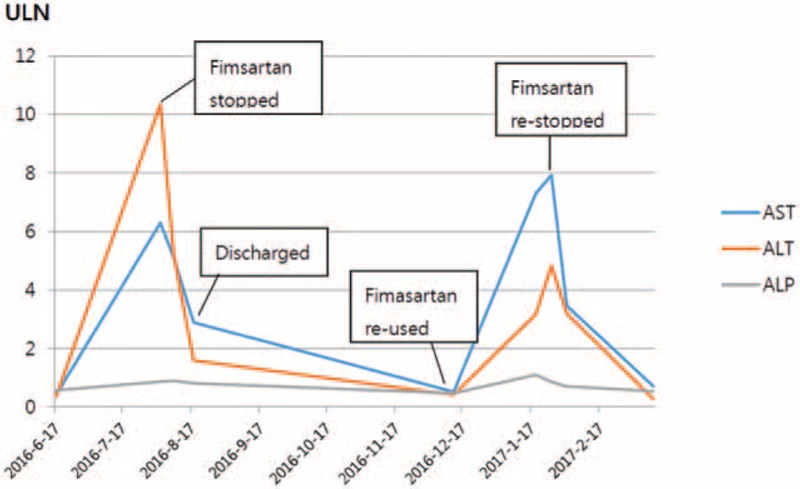
Courses of the laboratory findings during the first and second episodes of fimasartan-induced liver injury. ALP = alkaline phosphatase, ALT = alanine aminotransferase, AST = aspartate aminotransferase, ULN = upper limit of normal.

As a result, fimasartan was immediately discontinued and the patient was managed with supportive care via hepatotonics such as Godex (carnitine orotate). He showed improvement of the clinical and laboratory abnormalities, with AST and ALT levels of 44 and 34, respectively, after 3 weeks (on August 24, 2016) (Fig. 2).

He was re-referred from the local hospital due to re-elevation of liver enzymes on January 19, 2017. He was incidentally represcribed fimasartan 60 mg on December 22, 2016 at the local hospital. Fortunately, within a month after the patient discontinued using fimasartan, the liver enzyme level was normalized again (Fig. 2). This second episode strongly suggested the cause of toxic hepatitis was fimasartan.

## 4 Discussion

Fimasartan is a nonpeptide ARB. And it is used for heart failure or hypertension.^[9]^ Fimasartan blocks angiotensin II receptor type 1, and it reduces the prohypertensive actions of angiotensin II, through oral administration. Water retention by the kidneys and systemic vasoconstriction are inhibited.^[10]^ In several clinical trials, fimasartan was proofed the safety.^[1,9,11,12]^ Boryung Pharmaceuticals was approved by Korea Food and Drug Administration for fimasartan in September 2010 and launched the product in March 2011 as the brand name Kanarb, and the company is seeking a worldwide partnership for this drug.^[12]^ Nowadays, fimasartan is used in 13 countries including Russia, Singapore, and Latin Americas. And in a few years, fimasartan is used in more than 30 countries, including China. In clinical trials, fimasartan had a good safety profile and well tolerated, However, administration of higher dose (360 mg/day) was associated with increased incidences of postural dizziness and headache.^[9]^ In addition, in a small number of patients, adverse events such as syncope, cold feet, and diarrhea also appeared, and all of which eventually resolved without medical intervention.^[1,9]^

Drug-induced liver injury was used to be a uncommon problem, and each drugs has its own pattern of liver injury.^[13,14]^ Drug-induced liver injury can be classified as hepatocellular, cholestatic, or mixed types.^[15]^ In hepatocellular types, aminotransferase levels may be at least 5 times as high as normal. But in cholestatic types, elevations of ALP and bilirubin levels predominate.^[14]^ Liver biopsy may help identify the pattern of drug-induced liver injury in particular cases.^[16]^

Currently, drug-induced liver injury associated with ARBs use was reported in 18 cases: 6 with losartan use, 5 with irbesartan use, 4 with candesartan use, and 3 with valsartan use. The most common pattern of liver injury was hepatocellular; however, cholestatic and mixed patterns were also observed. The mechanism of liver injury was not well defined, but mostly resembled an idiosyncratic reaction. The onset of hepatotoxicity was variable, consisting with idiosyncratic reactions and arising as early as 8 days and up to 6 months after the initiation of therapy with an ARB. Normalization of liver enzymes took an average of 2 to 4 months after discontinuation of the drug.^[2–6,17,18]^

In our case, the patient had a hepatocellular pattern of injury in both first and second episode that was evident by the marked elevation of aminotransferases, reaching around 10 times the upper normal limit, with a disproportionate minimal elevation in bilirubin and ALP. Any extrahepatic obstruction was therefore unlikely. Other causes, such as viral hepatitis, autoimmune hepatitis, alcoholic hepatitis, acetaminophen toxicity, Wilson disease, malignancy, and sepsis that could cause liver damage resulting in a significant elevation of aminotransferase were excluded because of the patient's presentation during diagnostic investigations. After fimasartan was discontinued, the patient's liver enzymes were normalized within 3 weeks. Furthermore, after the second administration of fimasartan, the patient's liver enzymes rose more quickly than the first, and the liver enzymes were normalized after the patient stopped the fimasartan again. The modified RUCAM score of the second episode was 11, and all of this strongly indicated that the liver injury in our patient was due to fimasartan.

There are 3 interesting points in our case. First, the patient had taken 3 different types of ARBs before taking fimasartan, but these medications had no side effects. Second, drug-induced hepatotoxicity occurred in this patient only 10 months after the first dose of the drug, which is later than in other case reports. Finally, fimasartan was not initially thought to be a causative agent of hepatotoxicity, because it had been taking it for quite some time before the first hepatotoxicity occurred. After normalization of the patient's liver function, fimasartan was accidentally readministered, which caused the hepatotoxicity to be reproduced, making it clear what the causative agent was. Our case report has 2 instructive points. First, liver function tests should be performed periodically after the administration of fimasartan, and if hepatotoxicity is caused by it, it may be expressed later than other ARBs, which may occur even after 10 months. Since 2012, 3760 patients were prescribed fimasartan in our hospital, 2506 of them were prescribed for more than 3 months and 1636 were prescribed for more than 10 months. None of the other cases had hepatotoxicity due to fimasartan. Thus, hepatotoxicity caused by fimasartan is rare and should be taken more careful as it may appear late. Second, fimasartan-induced liver injury may occur in patients who have not had hepatotoxicity while taking other types of ARBs. We cannot explain why only fimasartan caused liver damage, although other ARBs had no problems in this patient. However, the small differences in the molecular structures of ARBs are thought to cause this phenomenon.

In summary, this case report described a 73-year-old man with hypertension who experienced liver injury after fimasartan administration. To our knowledge, there have been no published case reports about hepatotoxicity caused by fimasartan. Our case emphasizes that after initiation of fimasartan dosing, periodic liver function tests should be performed, even if the patient has been taking other types of ARBs without any problems.
